# Comparative analysis of kidney transplant costs related to recovery of renal function after the procedure

**DOI:** 10.1590/2175-8239-JBN-2020-0172

**Published:** 2021-04-23

**Authors:** Raquel Martins e Quinino, Fabiana Agena, Flávio Jota de Paula, William Carlos Nahas, Elias David-Neto

**Affiliations:** 1 Universidade de São Paulo, Faculdade de Medicina, Hospital das Clínicas, Serviço de Transplante Renal, São Paulo, SP, Brasil.

**Keywords:** Kidney Transplantation, Delayed Graft Function, Economics, Pharmaceutical, Transplante de Rim, Função Retardada do Enxerto, Farmacoeconomia

## Abstract

**Introduction:**

The number of kidney transplants (KTx) is increasing in Brazil and, consequently, the costs of this procedure increase the country's health budget. We retrospectively evaluated the data of kidney transplant procedures until hospital discharge, according to kidney function recovery after the procedure.

**Methods:**

Retrospective analysis of the non-sensitized, 1st KTx from deceased donors performed between Jan/2010 to Dec/2017.

**Results:**

Out of the 1300 KTx from deceased donors performed in this period, 730 patients were studied and divided into 3 groups: Immediate Renal Function (IRF) - decrease in serum creatinine ≥ 10% on two consecutive days; Delayed Graft Function (DGF) - decrease in serum creatinine <10% on two consecutive days, without the need for dialysis, and Dialysis (D) - need for dialysis during the first week. Patients in group D stayed longer in the hospital compared to DGF and IRF (21, 11 and 8 days respectively, p < 0.001). More D patients (21%) were admitted to the ICU and performed a greater number of laboratory tests (p < 0.001) and renal biopsies (p < 0.001), in addition to receiving a higher amount of immunosuppressants. Total hospital costs were higher in group D and DGF compared to IRF (U$ 7.021,48; U$ 3.603,42 and U$ 2.642,37 respectively, p < 0.001).

**Conclusion:**

The costs of the transplant procedure is impacted by the recovery of kidney function after the transplant. The reimbursement for each of these different kidney function outcomes should be individualized in order to cover their real costs.

## Resumo

**Introdução:**

O número de transplantes renais (KTx, do inglês kidney transplant) está aumentando no Brasil e, consequentemente, os custos deste procedimento aumentam o orçamento de saúde do país. Avaliamos retrospectivamente dados dos procedimentos de transplantes renais até a alta hospitalar, de acordo com a recuperação da função renal após o procedimento.

**Métodos:**

Análise retrospectiva dos 1º KTx de doadores falecidos, não sensibilizados, realizados entre Jan/2010 a Dez/2017.

**Resultados:**

Dos 1300 KTx de doadores falecidos realizados neste período, 730 pacientes foram estudados e divididos em 3 grupos: Função Renal Imediata (FRI) - diminuição na creatinina sérica ≥ 10% em dois dias consecutivos; Função Retardada do Enxerto (FRE) - diminuição na creatinina sérica <10% em dois dias consecutivos, sem necessidade de diálise, e Diálise (D) - necessidade de diálise durante a primeira semana. Pacientes no grupo D permaneceram mais tempo no hospital em comparação com FRE e FRI (21, 11 e 8 dias dias respectivamente, p < 0,001). Mais pacientes do grupo D (21%) foram admitidos na UTI e realizaram um maior número de testes laboratoriais (p < 0,001) e biópsias renais (p < 0,001), além de receberem uma quantidade maior de imunossupressores. Os custos hospitalares totais foram mais elevados nos grupos D e FRE em comparação com FRI (U$ 7.021,48; U$ 3.603,42 e U$ 2.642,37 respectivamente, p < 0,001).

**Conclusão:**

Os custos do procedimento de transplante são impactados pela recuperação da função renal após o transplante. O reembolso para cada um desses diferentes desfechos da função renal deve ser individualizado a fim de cobrir seus custos reais.

## Introducion

The number of kidney transplants (KTx) is increasing in Brazil and consequently the costs of this procedure increase the country's health budget. At the same time, the extended criteria donors are proportionally used more frequently to match the increased renal transplant demand. As consequence, there is an increase in the number of patients needing dialysis after transplantation with longer hospital stays.

In countries like Brazil, where hospital reimbursement of the medical procedures is fixed for each procedure and defined by national health system, (Sistema Único de Saúde-SUS in Brazil), the variations in clinical outcomes may impact hospitals' budget. Adjusting these fixed costs, according to the renal function outcome immediately after transplant, should be a demand from hospitals. On the other side, demonstrating these different costs is mandatory to convince the health authorities of the needed reimbursements adjustments.

The use of a perfusion machine instead of cold storage to diminish the need for dialysis after transplant (Tx) increases the costs of the transplant procedure. However, this increased cost will be welcomed if the final costs of KTx is reduced even with the added costs of the machine perfusion.

Similarly, to analyze dialysis after transplantation, it is mandatory to verify whether, and by how much, dialysis after KTx impacts the total costs of the procedure.

In this study, we retrospectively evaluated the hospital costs of KTx procedure in a homogenous group of non-sensitized patients who performed their 1^st^ KTx from a deceased donor.

## Methods

We retrospectively evaluated all deceased-donor KTx performed at our center between Jan/2010 to Dec/2017. The exclusion criteria were: children (< 18 years), re-transplants, other simultaneous solid organ transplants (SOT), and sensitization (patients with PRA class I or II > 10%).

Data were collected from hospital admission until hospital discharge.

Dialysis sessions were performed as clinically indicated and according to the physician discretion. We defined the following groups according to the kidney function after KTx:

*1-Immediate kidney function (IRF)*: A decrease in serum creatinine ≥ 10% in two consecutive days, (1^st^ to 2^nd^ and/or 2^nd^ to 3^rd^).

*2-Delayed Graft Function (DGF)*: A decrease in serum creatinine < 10% in two consecutive days, (1^st^ to 2^nd^ and/or 2^nd^ to 3^rd^) but without the requirement of dialysis in the first week.

*3-Dialysis (Dialysis)*: Requirement of dialysis during the first week. Patients who were submitted to dialysis immediately after transplant or in the first post-operative day due to hypervolemia, hyperkalemia, or other causes but were not submitted to other dialysis sessions in the first week were not included in this group but were included in the DGF group.

Hospital costs were analyzed according to the number of procedures and the number of days in hospital.

The costs were calculated according to the Hospital das Clínicas - Faculdade de Medicina da Universidade de São Paulo (HCFMUSP) table of costs, which estimates the daily ward cost per day as U$ 320.00 and the ICU cost per day as U$ 378.00. To calculate costs of immunosuppressive drugs we retrieved the exact amount of each immunosuppressive drug (in mg/day) received during the hospital stay of each patient and multiplied the amount of drugs in mg by days in the hospital and by the mg cost of the drug acquisition by the hospital.

Data is presented as mean ± SD. To evaluated differences in the proportion of categorical variables Q-square was used. For continuous variables, one-way ANOVA and ANOVA on Ranks (with Dunn's method) were used. Results were analyzed using the statistical software package SPSS (version 18.0; SPSS Inc., Chicago, IL).

## Results

**Figure 1:**
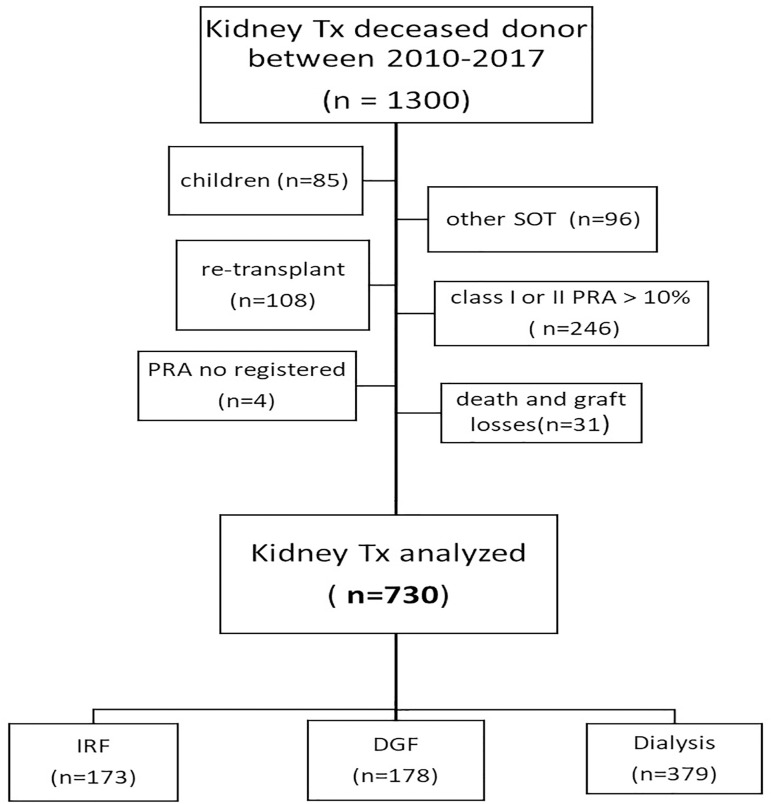
Study flowchart.

Figure 1 shows the flowchart of the study. From January 2010 to December 2017, 1300 kidney transplants were performed at our center. Of these, 539 were excluded: children (n = 85); re-transplants (n = 108), other SOT (n = 96) and PRA class I or II > 10% (n = 246). Four patients without a registered PRA were also excluded.

In addition, from the group of 761 selected patients, all deaths until the seventh day (n = 17), all graft losses until the second postoperative day (n = 9; as 1 hypo-perfused kidney, 1 renal vein laceration, 2 venous thromboses and 5 arterial thromboses) and losses from the second to the seventh postoperative day due to thrombosis probably related to the surgical technique (n = 5) were also excluded, resulting in 730 KTx analyzed.

These 730 patients were then divided into the three groups (IRF n = 173, 23.7%), (DGF n = 178, 24.4%), (Dialysis n = 379, 51.9%).

Table 1 shows the demographics, native kidney diseases, and transplant features of the groups.

**Table 1:** Recipients features

Group	IRF n = 173	DGF n = 178	Dialysis n = 379	p value
Gender (F/M), n	71/102	66/112	139/240	0.602
Age (y) ± SD	50 ± 14	50 ± 13	53 ± 13	0.05
Race (white/non-white/other), n	110/58/05	129/48/01	257/114/08	0.297
Cause of chronic renal disease				
Unknown CKD	48	40	89	
CGN	33	39	84	
Diabetic nephropathy	37	39	108	
Hypertensive nephrosclerosis	29	23	42	0.105
ADPKD/ALPORT	13	15	25	
T-I nephritis	6	10	22	
Urological congenital	5	4	6	
Others	2	8	3	
Type of dialysis				
HD	148	156	362	< 0.001
PD	24	19	11	
No dialysis	1	3	6	
Time on dialysis (mo) median (25-75%)	37 (22-62)	32 (19-48)	45 (27-74)	< 0.001
**Transplant data**				
Induction therapy				
ATG/Basiliximab	64/107	52/125	131/246	0.492
Baseline immunosuppression				
Tacrolimus	170	177	374	0.932
MPA	168	176	375	

CKD: chronic kidney disease; CGN: chronic glomerulonephritis; ADPKD: autosomal dominant polycystic kidney disease; T-I nephritis: tubulo-interstitial nephritis; ATG: anti-thymocyte globulin; MPA: mycophenolic acid; TAC: tacrolimus.

In the Dialysis group (D group), recipients were older than in the other groups. Less patients in the D group were under peritoneal dialysis before transplantation. Time on dialysis was also longer in the D group as compared to the other two groups. Only a few patients did not receive tacrolimus (TAC) or mycophenolic acid (MPA). All patients received oral prednisone.

Table 2 shows the donor data. The donors were older in the D group as compared to IRF. More patients in the D group received kidneys from expanded criteria donors as compared to DGF and IRF (33%, 25%, 20%, respectively), and the difference was statistically significant between IRF and D groups (*p*= 0.04) with a trend between DGF and D (*p* = 0.09). The Kidney Donor Risk Index (KDRI) was progressively higher from IRF to DGF and D. Also, the cold ischemia time was longer for the D group.

**Table 2:** Donor features

Group	IRF n = 173	DGF n = 178	Dialysis n = 379	*p* value
Gender F/M, n	69/104	79/99	148/231	0.480
Age (y), ± SD	41 ± 14	46 ± 13	48 ± 13	0.000
Race (white/non-white/other/unknown)	93/75/1/4	100/72/2/4	207/155/6/11	0.953
KDRI median (range)	1.03 (0.80-1.28)	1.11 (0.94-1.46)	1.2 (1.00 ±1.50)	<0.001
Cold ischemic time,(h) mean±SD	26 ± 6	26 ± 6	27 ± 6	0.017
Donor type (SCD/ECD/NR)	126/31/16	128/43/7	233/113/33	0.008
ECD	31(20%)*	43(25%)^&^	113(33%)	
Perfusion solution				
Euro-Collins/ Belzer type solution/ NR	139/33/1	131/44/3	302/76/1	0.196

KDRI: kidney donor risk index; SCD: standard criteria donor; ECD: extended criteria donor; NR: not registered. *P* = 0.04 -

* IRF x Dialysis; *P* = 0.09 -

& DGF x Dialysis

Table 3 shows the number of days in hospital, the percentage of patients admitted to intensive care unit (ICU), and number of days in ICU after the KTx procedure, as well as the estimated glomerular filtration rate (eGFR) at hospital discharge.

**Table 3:** Days in hospital for TX, days in ICU, and GFR at discharge

Group	IRF n = 173	DGF n = 178	Dialysis n = 379	*p* value
Days in hospital (mean ± SD)	8 ± 6	11 ± 6	21 ± 15	< 0.001
Number of patients in ICU, (%)	16 (9%)	13 (7%)	79 (21%)	< 0.001
Days in ICU (mean ± SD)	2 ± 1	2 ± 1	5 ± 6	0.025
GFR at hospital discharge (mL/min/1.73m^2^) (mean± SD)	25 ± 14	14 ± 10	14 ± 14	< 0.001

ICU: intensive care unit; eGFR: estimated glomerular filtration rate.

Patients in D group stayed an average of 10 days more in the hospital compared to IRF and DGF (*p* < 0.001) groups. More patients (21%) of the D group were admitted to an ICU compared to IRF (9%) and DGF (7%) and stayed more days in the ICU (*p* = 0.025). Patients in the IRF group were discharged with eGFR (estimated glomerular filtration rate)higher than the DGF and D groups (*p* < 0.001).

Table 4 shows the number of laboratory tests performed during hospital stay.

**Table 4:** Number of laboratory tests performed per patient in each group

Group	IRF n = 173	DGF n = 178	Dialysis n = 379	*p* value
Total blood counts (mean ± SD)	8 ± 5*	11 ± 6**	19 ± 14* ^,&^	< 0.001
Serum creatinine (mean ± SD)	8 ± 5*	12 ± 7**	20.7 ± 14.5* ^,&^	< 0.001
Urinary Prot/Creat (mean ± SD)	2.1 ± 1.3^&^	2.7 ± 1.8**	4.5 ± 3.3^&,^ **	< 0.001
AST (mean ± SD)	2.9 ± 2.0^&^	3.9 ± 3.0**	7.0 ± 6.3^&,^ **	< 0.001
Tacrolimus blood level (mean ± SD)	2.5 ± 1.4	3.6 ± 2.3	6.4 ± 4.3	< 0.001

P <0.05 -

* IRF x DGF;

& IRF x Dialysis;

** DGF x Dialysis AST: Aspartate aminotransferase.

Patients from the D group performed a higher number of tests, including blood counts, serum creatinine, urinary protein/creatinine, aspartate aminotransferase (AST), and tacrolimus dosage (*p* < 0.001).

Table 5 shows the number of imaging exams and renal biopsies by group and the mean number of patients submitted to the procedures.

**Table 5:** Exams performed by group and per patient

Group		IRF n = 173	DGF n = 178	Dialysis n = 379	*p* value
Renal Biopsies	n (%)	105 (61%)	112 (63%)	280 (74%)	
Per patient	mean ± SD	1.1 ± 0.39	1.15 ± 0.41	1.6 ± 0.77	< 0.001
Allograft USound/ Dopller	n (%)	151 (87%)	144 (81%)	310 (82%)	
Per patient	mean ± SD	1.7 ± 0.94	1.97 ± 1.19	1.78 ± 1.19	0.071
X- Ray	n (%)	72 (42%)	63 (35%)	154 (41%)	
Per patient	mean ± SD	2.92 ± 4.53	3.37 ± 4.49	3.58 ± 5.78	0.544
Echocardiogram	n (%)	24 (14%)	24 (13%)	42 (11%)	
Per patient	mean ± SD	1.04 ± 0.20	1.13 ± 0.45	1.31 ± 0.84	0.253
CT abdomen	n (%)	16 (9%)	20 (11%)	55 (14%)	
Per patient	mean ± SD	1.69 ± 1.54	1.25 ± 0.55	1.35 ± 0.82	0.888
CT chest	n (%)	6 (3%)	5 (3%)	13 (3%)	
Per patient	mean ± SD	1.17 ± 0.41	1.00 ± 0.00	1.23 ± 0.44	0.515
US abdomen	n (%)	6 (3%)	7 (4%)	18 (5%)	
Per patient	mean ± SD	1.0 ± 0.0	1.71 ± 1.11	1.17 ± 0.51	0.083

n(%)= number of patients/percentage of total; mean ± SD of procedures performed Per patient: the mean number of exam per patient out of those who performed it.

More patients in the D group were submitted to a renal biopsy and those patients performed a higher number of biopsies.

Due to the fact that the exact number of dialysis sessions performed until patient discharge could not be counted in all patients due to possible missing data after the first week, the number of dialysis performed by the D group was analyzed only for patients transplanted in 2016-2017 (n = 76) when all dialysis sessions were registered in the electronic data set.

The total number of dialysis sessions for these patients was 196. Mean number of dialysis sessions was 2.6/patient; 34% of the patients required only one more dialysis session after the one performed immediately after surgery or 1^st^ post-operative day. The other 66% of the patients required 2 or more dialysis sessions after KTx.

Table 6 describes the costs of immunosuppressive drugs according to the acquired drug costs of HCFMUSP. There was a strong correlation between days of hospitalization and total doses of mycophenolate sodium (MPS) and TAC (data not shown). As expected, the longer the stay, the greater the amount of immunosuppressants given to each patient. Drug costs followed the same trend. The IRF group presented lower stay, lower dose of immunosuppressants, and lower costs. The DGF group had intermediate numbers and the D group had longer stay and higher immunosuppressants cost, with statistical significance. The dose of ATG (anti-thymocyte globulin) used in induction did not differ among groups.

**Table 6:** Immunosuppressive drugs costs (in U$) during hospital stay

Group	IRF	DGF	Dialysis	*p* value
MPS (mg)	9,153.35 ± 5,465.73	12,744.46 ± 7,518.15	21,402.13 ± 14,068.18	< 0.001
Costs (U$)	104.01 ± 66.01	147.21 ± 89.64	247.49 ± 167.00	< 0.001
**TAC** (mg)	79.37 ± 51.75	117.41 ± 74.69	197.13 ± 131.85	< 0.001
Costs(U$)	142.57 ± 98.24	215.97 ± 140.02	361.80 ± 247.16	< 0.001
**ATG** (mg)	225.15 ± 133.96	215.00 ± 125.22	280.22 ± 180.47	0.102
Costs(U$)	4,597.05 ± 2,735.30	4,389.86 ± 2,556.79	5,721.55 ± 3,684.92	0.102

MPS: mycophenolate sodium; TAC: tacrolimus; ATG: thymoglobulin.

Table 7 shows the costs of hospitalization at our institution. The costs were proportional to days in hospital, i.e., highest in the D group, intermediate in DGF, and lowest in the IRF group. The same also occurred with days of ICU stay. The increase in hospital costs is also much higher for patients who need an ICU care, where the reimbursement by the government is much less than what is necessary to cover the costs.

**Table 7:** Total hospital ward costs (in U$) in each group

Group	IRF	DGF	Dialysis	*p* value
Costs for the days in hospital (U$)	2,451.29 ± 1,732.18* ^&^	3,407.36 ± 2,009.63^§^	6,350.77 ± 4,753.59	< 0.001
Costs for the days in ICU (U$)	897.60 ± 495.14* ^&^	959.45 ± 396.76^§^	2,095.54 ± 2,253.10	0.003
Total costs (U$)	2,642.37 ± 1,850.46* ^&^	3,603.42 ± 2,107.48^§^	7,021.48 ± 5,505.75	< 0.001

P < 0.05 -

* IRF x DGF;

& IRF x Dialysis;

§ DGF x Dialysis.

## Discussion

In this retrospective analysis we have shown that there are different costs for the KTx procedure, from hospital admission to discharge, depending upon the recovery of renal function after transplantation. This recovery time directly correlates with the number of days in hospital and directly impacts total costs.

The data showed that not only the dialysis patients but also those who developed DGF without need of dialysis, stayed longer in hospital and their costs surpassed the government reimbursement for the transplant procedure. In a recent publication by Kim et al.^1^, similar results were demonstrated. However, to our knowledge this is the first large analysis of costs for this specific transplant population, in Brazil. These figures should open a discussion with the SUS for a differential reimbursement for the three categories of renal function outcome. They also provide support for hospital administrators to negotiate with private health insurances companies different costs for payment according to the post-transplant immediate renal function.

We have selected a large homogeneous population of non-sensitized adult recipients, receiving their first transplant from deceased donors in order to avoid the impact of sensitization^2^^,^ 3 and re-transplant^4^ in cost outcome. With this selected population, all costs including blood tests, immunosuppressive drugs, image exams, biopsies, etc., could be evaluated and all of them directly correlated with the number of days in the hospital.

We observed that 52% of our patients required dialysis sessions in the first week post-transplant. This figure is much lower than the 70-75% of dialysis need, frequently described in the Brazilian population 5 ^,^ 6 ^,^ 7 ^,^ 8 ^,^ 9 ^,^ 10, possibly because of the low-risk population selected for this study and because we did not count dialysis sessions done only in the first post-operative day. The need for immediate dialysis but no further ones in the first week is frequently due to hypervolemia and hyperkalemia and do not reflect the status of kidney function after transplantation^11^^,^ 12. Instead, we classified patients in dialysis group if they required at least another dialysis session in the first week.

However, all the above-mentioned studies compared patients who required dialysis versus those who did not, without considering a subgroup of patients who did not require dialysis but still did not have adequate kidney function to be discharged from the hospital, here classified as DGF group. In our analysis, this group of patients differed completely from those with immediate renal function in terms of costs. They represent a separate group who stayed longer in the hospital until they recover enough kidney function to be discharged. The rationale for this separation is that they were discharged from the hospital with an eGFR of only 14mL/min/1.73m^2^, the same function that the D group was discharged with.

The second question is whether there are changes that we can make to decrease the rate of patients who need dialysis after transplantation. Variables such as time and type of pre-transplant dialysis, age of donor, and expanded criteria donor are not modifiable, and therefore, the only modifiable factor may be the organ preservation after harvesting.

There are many reports showing that the perfusion machine reduces the incidence of DGF, the duration of DGF, the length of hospital stay^5^^,^ 7 ^,^ 13 ^,^ 14 ^,^ 15, and the costs related to transplantation, in addition to the better cost-effectiveness of the perfusion machine as compared to cold storage^16^^,^ 17 ^,^ 18 ^,^ 19 ^,^ 20 ^,^ 21.

In our hospital, one day in the hospital costs around U$ 320.00. The total cost for preservation in a perfusion machine in Brazil is approximately U$ 2,200.00 (approximately the cost of 6 days in the hospital), according to data from the manufacturer. Therefore, this procedure will only have large acceptance if it can decrease the number of hospital days to 6 days or less, just to be even to the current costs of longer stays in the hospital.

In our opinion, the major problem of perfusion machine studies is that they included all recipients from deceased donors during the study period, and therefore including those who would never require dialysis. On the other side, if we treat in a perfusion machine only kidneys who have a great chance of requiring dialysis we will spend money with the perfusion machine in only 52% of the patients. This policy may change the economics behind using perfusion machines in renal transplantation.

The real cost-benefit of this procedure in reducing dialysis need can only be evaluated with studies that include patients with high risk for dialysis after transplantation, like those receiving kidneys with longer cold ischemia time, higher KDRI, from expanded criteria donors, etc. Developing a high sensitivity and specificity equation to identify these patients is our future purpose.

In conclusion, we have shown differences in costs of KTx in low-risk patients depending on the recovery of kidney function after transplantation. It seems that the most viable form to reduce these costs is to implement ways for better organ preservation. However, the cost-benefit, cost-effectiveness, and feasibility of this idea remains to be determined.
